# Phase 1 study of oral TAS-102 in patients with refractory metastatic colorectal cancer

**DOI:** 10.1007/s00280-015-2850-4

**Published:** 2015-09-14

**Authors:** Johanna C. Bendell, Lee S. Rosen, Robert J. Mayer, Jonathan W. Goldman, Jeffrey R. Infante, Fabio Benedetti, Donghu Lin, Hirokazu Mizuguchi, Christopher Zergebel, Manish R. Patel

**Affiliations:** Sarah Cannon Research Institute/Tennessee Oncology, PLLC, 250 25th Avenue North, Suite 200, Nashville, TN 37203 USA; University of California, 2020 Santa Monica Blvd., Suite 600, Los Angeles, CA 90404 USA; Dana-Farber Cancer Institute, 450 Brookline Ave., Dana 1602, Boston, MA 02215 USA; Taiho Oncology Inc., 212 Carnegie Center, Suite 201, Princeton, NJ 08540 USA; Taiho Pharmaceutical of Beijing Co., Ltd., 9F-9A3, Hanwei Plaza, 7th, Guanghua Rd., Chaoyang Dist., Beijing City, 100004 China; Sarah Cannon Research Institute/Florida Cancer Specialists, 600 N. Cattlemen Road, Suite 200, Sarasota, FL 34232 USA

**Keywords:** Antimetabolite, Metastatic colorectal cancer, Phase 1 study, Recommended dose, Safety, TAS-102

## Abstract

**Purpose:**

To evaluate safety of TAS-102 administered twice daily (bid) on days 1–5 and 8–12 of a 4-week cycle, confirm feasibility of the Japanese recommended dose (RD), 35 mg/m^2^, in Western patients with metastatic colorectal cancer (mCRC) refractory to standard chemotherapies, and describe preliminary antitumor activity.

**Methods:**

This open-label, dose-escalation phase 1 study was conducted at four US centers. Patients were enrolled into two sequential cohorts [30 (cohort 1) or 35 mg/m^2^/dose bid (cohort 2)]; dose-limiting toxicities (DLT) were evaluated during cycle 1 in dose-escalation cohorts. At RD, 15 additional patients were enrolled in an expansion cohort.

**Results:**

Patients (*N* = 27) with refractory mCRC received TAS-102; 74 % had received ≥4 prior regimens. DLT was not observed in three patients in cohort 1, and was in one out of nine patients in cohort 2 (grade 3 febrile neutropenia). Therefore, RD was identified as 35 mg/m^2^ bid. At RD, fatigue (63 %), gastrointestinal disturbances and nausea (46 %), vomiting (46 %), and diarrhea (42 %) were common but rarely grade 3/4. Grade 3/4 nausea, vomiting, and diarrhea occurred at 4 % each. Grade 3/4 toxicity was predominantly hematologic [neutropenia (71 %), anemia (25 %)]; febrile neutropenia was observed in two patients. Stable disease lasting ≥6 weeks was achieved by 16 evaluable patients (70 %); median progression-free survival and overall survival were 5.3 and 7.5 months, respectively.

**Conclusions:**

TAS-102 has an acceptable safety profile and preliminary evidence of disease stabilization in Western patients with refractory mCRC. Results from a randomized phase 3 study have shown survival benefit with disease stabilization evidence in this population.

## Introduction

Colorectal cancer is the third most common malignancy and the second leading cause of cancer death in men and women in the USA [1, 2]. Fifty to sixty percent of patients diagnosed with colorectal cancer will develop metastatic disease [3]. For patients with unresectable metastatic colorectal cancer (mCRC), initial treatment typically consists of an infusional 5-fluorouracil (5-FU) and leucovorin regimen containing irinotecan (FOLFIRI), oxaliplatin (FOLFOX), or both (FOLFOXIRI) [3–6]. Use of 5-FU, irinotecan, and oxaliplatin, regardless of their sequence in frontline and second-line treatment, is associated with significantly improved survival [7]. The addition of monoclonal antibodies targeting vascular endothelial growth factor (bevacizumab) or epidermal growth factor receptor (cetuximab or panitumumab) to chemotherapy has also led to improved outcomes [8–10]. In the refractory setting, regorafenib has shown modest improvement in overall survival (OS) compared with placebo [11]. More treatment options are needed for patients with disease progression after 5-FU, irinotecan, oxaliplatin, and available monoclonal antibodies.

TAS-102 is a novel, orally active, antineoplastic combination drug consisting of trifluridine (FTD) and tipiracil hydrochloride (TPI) in a molar ratio of 1:0.5. FTD is a thymidine-based nucleoside analogue, which, in its triphosphate form, is incorporated into DNA causing single-strand and double-strand breaks (TS) [12, 13]. TPI is a potent inhibitor of thymidine phosphorylase, which prevents rapid degradation of FTD, thereby allowing sustained FTD concentrations to be maintained after oral administration [14]. The incorporation of FTD into DNA appears to be the primary mechanism of antitumor activity of TAS-102 compared with 5-FU and other fluoropyrimidines that produce cytotoxic effects primarily via TS inhibition [15]. Consistent with this difference, TAS-102 antitumor effects have been observed in colorectal cancer cell lines and xenograft models resistant to 5-FU and other fluoropyrimidines [16, 17]. In preclinical models, TAS-102 induces higher levels of apoptosis compared with 5-FU and, unlike 5-FU, does not elicit an autophagic survival response in colorectal cancer cell lines [18].

Treatment on days 1–5 and 8–12 of a 4-week cycle was chosen as the recommended phase 2 schedule for TAS-102 administration based on phase 1 clinical studies in patients with advanced solid tumors [19–21]. In Japanese patients, most of whom had mCRC, the maximum tolerated dose (MTD) on this schedule was 35 mg/m^2^/dose twice daily [22].

However, a previous phase 1 study conducted in the USA in heavily pretreated patients with breast cancer identified 25 mg/m^2^/dose twice daily as the MTD [23], although higher daily doses were found to be tolerable with other dosing schedules in patients with other advanced tumors, particularly gastrointestinal tumors [21]. Therefore, the present study was designed to evaluate the safety and determine whether the dose and schedule of TAS-102 used in the Japanese mCRC phase 2 trial is feasible in similar patients with refractory mCRC in the USA, and to obtain preliminary information about its antitumor activity.

## Methods

### Patients

Male or female patients aged ≥18 years of age with histologically or pathologically confirmed refractory mCRC were eligible if they had received at least two prior lines of conventional chemotherapy for metastatic disease, including a fluoropyrimidine, oxaliplatin, and irinotecan, had Eastern Cooperative Oncology Group (ECOG) performance status 0 or 1, were able to take medications orally, had adequate organ function, and had a life expectancy of at least 3 months. Adjuvant therapy with a fluoropyrimidine and oxaliplatin could count as one line of prior therapy if disease recurred within 12 months of completion. Previous treatment with biologics targeting vascular endothelial growth factor, epidermal growth factor, or their receptors was allowed. Patients with known brain metastasis, acute systemic infection, grade ≥3 ascites, or significant cardiovascular disease; those who underwent major surgery or radiotherapy within the previous 4 weeks, had radiation to >25 % of marrow-bearing bone, or had received anticancer therapy within 3 weeks; and women who were pregnant or lactating, were excluded.

### Study design

This open-label, non-randomized, sequential-group, dose-escalation phase 1 study was conducted at four centers in the USA from September 2011 to April 2013. The data cutoff date was April 3, 2013, which was 12 months after the last enrolled patient started treatment. The study was conducted in accordance with ethical principles originating in the Declaration of Helsinki (2008) and in compliance with Good Clinical Practice and all local and national regulatory guidelines. Study approval was obtained from the institutional review board at each site before that site enrolled any patients. All patients provided written informed consent before any screening procedures were conducted.

Eligible patients were assigned sequentially to two cohorts corresponding to TAS-102 doses of 30 mg/m^2^/dose twice daily (cohort 1) or 35 mg/m^2^/dose twice daily (cohort 2) on days 1–5 and 8–12 of each 4-week cycle. Doses were taken orally within 1 h after completing morning and evening meals.

Three patients were assigned initially to the first dose level using a conventional 3 + 3 dose-escalation design. If a dose-limiting toxicity (DLT) occurred during the first cycle in ≥2 patients, the study was to be stopped. If DLTs were not observed in the first three patients (or in ≤1 of 6 patients), then six patients were to be enrolled at the second dose level, and additional patients were allowed to be enrolled in any cohort at the discretion of the investigator and sponsor if further safety evaluation was warranted. DLTs were defined as grade 4 neutropenia lasting >7 days, febrile neutropenia (absolute neutrophil count <1000/mm^3^ with a single body temperature of >38.3 °C [101 °F] or sustained temperature of ≥38 °C [100.4 °F] for more than 1 h), grade 4 thrombocytopenia or grade 3 thrombocytopenia with bleeding, grade 3/4 nausea or vomiting lasting >48 h and uncontrolled by aggressive antiemetic therapy, grade 3/4 diarrhea lasting >48 h and unresponsive to antidiarrheal medication, other grade 3/4 non-hematologic toxicity, any drug-related toxicity resulting in >1 week delay in initiation of cycle 2, or any drug-related toxicity preventing administration of ≥80 % of the planned cumulative dose during cycle 1. The recommended dose (RD) for further clinical development was defined as the highest dose level at which <33 % of patients experienced a DLT during the first cycle. After establishing the RD, additional patients were enrolled into the expansion cohort in order to confirm safety and assess preliminary antitumor activity.

TAS-102 treatment was discontinued if patients experienced disease progression, unacceptable toxicity, withdrew consent, or at the investigator’s discretion if felt to be clinically indicated. Study treatment was supplied as 15- and 20-mg immediate-release film-coated tablets; the number of tablets needed to achieve the assigned dose was determined according to the patient’s body surface area. Dose reductions were permitted in 5 mg/m^2^ decrements to a minimum dose of 20 mg/m^2^/dose in the event of treatment-related toxicity (grade ≥3 non-hematologic toxicity, grade 4 neutropenia, or grade ≥3 thrombocytopenia). Patients who did not meet resumption criteria within 14 days of the scheduled initiation of the next cycle were discontinued from treatment.

Other anticancer treatments, including palliative radiotherapy, were not allowed. Prophylactic administration of granulocyte colony-stimulating factor (G-CSF), antidiarrheal agents, or antiemetic agents were not permitted during cycle 1.

### Assessments

Patients were monitored for adverse events throughout the study and for up to 30 days after the last dose. Adverse events were graded according to the National Cancer Institute Common Terminology Criteria for Adverse Events (version 4.03) and coded using the Medical Dictionary for Regulatory Activities (version 14.0). Other safety assessments were made before each cycle and at the 30-day safety follow-up visit, including hematology and clinical chemistry (also on days 8, 15, and 22 of cycle 1 and days 1 and 22 of subsequent cycles), physical examination, vital signs, performance status, and electrocardiogram (also on day 12 of cycle 1).

Tumor assessments and imaging studies of the chest, abdomen, and pelvis as clinically indicated were obtained at baseline (within 28 days before first dose) and then every two cycles. *KRAS* status was determined locally during screening. Tumor response was determined by the investigator according to RECIST criteria (version 1.1) [24]. Patient survival was determined every 2 months after the end of treatment.

### Statistical analysis

The sample size was not based on formal hypothesis testing, but was selected to obtain sufficient safety data in order to identify the RD for future clinical studies.

Safety was evaluated in all patients who received at least one dose of TAS-102. DLTs were evaluated in all patients in cohorts 1 and 2 who received at least 80 % of the planned study medication during cycle 1 (or lesser amounts due to occurrence of a DLT). Efficacy was assessed in all patients who completed at least one cycle of treatment and had clinical or radiologic assessments for disease progression.

Statistical analyses were performed using SAS^®^ software (version 9.1; SAS Institute, Inc., Cary, NC). Safety parameters were summarized descriptively. Efficacy parameters were determined for all patients and by *KRAS* status. The best overall response was based on investigator assessment of radiologic images and classified as complete response (CR), partial response (PR), stable disease (SD), or progressive disease (PD) according to RECIST criteria (version 1.1) [24]. SD required disease maintenance for at least 6 weeks without evidence of progression. Disease control rate (DCR) was defined as the proportion of patients with objective evidence of CR, PR, or SD and was summarized descriptively. Progression-free survival (PFS, defined as the time from the first dose of TAS-102 until radiologic disease progression or death) and OS were evaluated by the Kaplan–Meier method, and 95 % confidence intervals (CI) for median survival values were determined by the methods of Brookmeyer and Crowley [25].

### Role of the sponsor

This study was sponsored, designed, and analyzed by Taiho Oncology, Inc. The investigators enrolled and treated the patients and collected the data. All authors had access to the data and vouch for its accuracy. A draft of the manuscript was written by a medical writer who was compensated by Taiho Oncology, Inc. The authors provided guidance for the draft, comments, and feedback on subsequent revised versions. The corresponding author made the final decision to submit the manuscript for publication.

## Results

### Patient disposition and characteristics

**Fig. 1:**
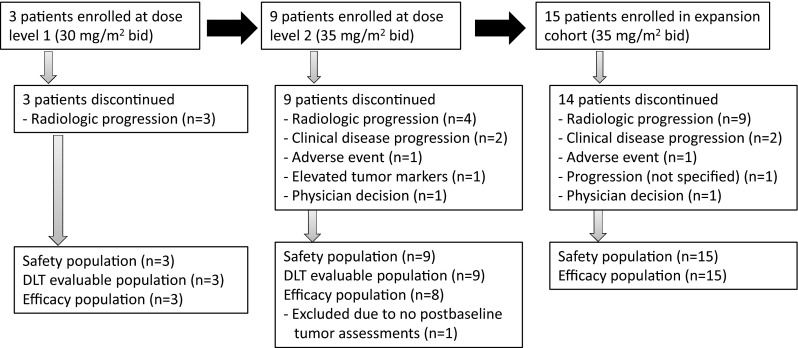
Patient disposition

A total of 27 patients received TAS-102, including three patients in cohort 1 (30 mg/m^2^/dose twice daily), nine patients in cohort 2 (35 mg/m^2^/dose twice daily), and 15 patients in the expansion cohort (35 mg/m^2^/dose twice daily) (Fig. 1). All treated patients from cohorts 1 and 2 were included in the DLT evaluable population, and all patients were included in the safety evaluation. One patient without any post-baseline tumor assessments was excluded from the efficacy evaluation. At the time of data cutoff, one patient in the expansion cohort remained on TAS-102 treatment. Most patients discontinued study treatment due to radiologic progression.

The study population had a median age of 64 years (range, 44–88), most were men (63 %) and white (93 %), and all had ECOG performance status of 0–1 (Table 1). The study population was heavily pretreated; the majority (74 %) had received ≥4 prior regimens, and besides a fluoropyrimidine, irinotecan, and oxaliplatin, most had received bevacizumab (96 %) and either cetuximab or panitumumab (52 %). *KRAS* status was determined for 25 patients [12 (48 %) wild-type and 13 (52 %) *KRAS* mutant].

**Table 1:** Patient demographics and baseline characteristics

Characteristic	Dose level 1 (30 mg/m^2^) (*n* = 3)	Dose level 2 (35 mg/m^2^) (*n* = 9)	Expansion cohort (35 mg/m^2^) (*n* = 15)	All patients (*N* = 27)
Median age, years (range)	55 (51–64)	48 (44–75)	68 (50–88)	64 (44–88)
Gender, *n* (%)				
Male	0	7 (77.8)	10 (66.7)	17 (63.0)
Female	3 (100)	2 (22.2)	5 (33.3)	10 (37.0)
Race, *n* (%)				
White	2 (66.7)	8 (88.9)	15 (100)	25 (92.6)
Black/African-American	1 (33.3)	1 (11.1)	0	2 (7.4)
ECOG performance status, *n* (%)				
0	3 (100)	6 (66.7)	7 (46.7)	16 (59.3)
1	0	3 (33.3)	8 (53.3)	11 (40.7)
Number of prior regimens, *n* (%)				
2	0	1 (11.1)	2 (13.3)	3 (11.1)
3	0	1 (11.1)	3 (20.0)	4 (14.8)
≥4	3 (100)	7 (77.8)	10 (66.7)	20 (74.1)
Prior therapy, *n* (%)				
Bevacizumab	3 (100)	8 (88.9)	15 (100)	26 (96.3)
Cetuximab, or panitumumab	1 (33.3)	6 (66.7)	7 (46.7)	14 (51.9)
Other agents^a^	3 (100)	9 (100)	14 (93.3)	26 (96.3)
*KRAS* status, *n* (%)				
Wild type	1 (33.3)	5 (55.6)	6 (40.0)	12 (44.4)
Mutant^b^	2 (66.7)	3 (33.3)	8 (53.3)	13 (48.1)
Not determined	0	1 (11.1)	1 (6.7)	2 (7.4)

*ECOG* Eastern Cooperative Oncology Group

^a^Agents other than a fluoropyrimidine, irinotecan, and oxaliplatin; none had previously received regorafenib

^b^Mutations were identified in codon 12 [including G12V (*n* = 5), G12D (*n* = 4), and G12S (*n* = 1)] and in codon 13 [G13D (*n* = 3)]

### Dose-limiting toxicity

DLT was not observed among the three patients who received TAS-102 at a dose of 30 mg/m^2^/dose twice daily; therefore, the dose was escalated to 35 mg/m^2^/dose twice daily. Among the first six patients in cohort 2, one patient had a DLT of grade 3 febrile neutropenia, which was considered related to study treatment and resolved during cycle 1. Three additional patients were enrolled in cohort 2 to confirm tolerability, and none had DLTs. Therefore, the RD was defined as 35 mg/m^2^/dose twice daily, and the expansion cohort was enrolled to obtain additional safety data at this dose.

### Exposure

Patients received TAS-102 for a median of three cycles (range, 1–14). Seven patients (26 %) initiated at least eight cycles of study treatment. Among the 24 patients who received 35 mg/m^2^ twice daily, the median cumulative dose was 2052 mg/m^2^ (range, 706–8949), median dose intensity was 154.2 mg/m^2^/week (range, 108.5–176.6), and relative dose intensity (i.e., ratio of actual to planned dose intensity) was 0.89 (range, 0.62–1.01).

There were no dose reductions in cohort 1, although two of the three patients had the start of a cycle delayed by ≥4 (but <8) days. Ten patients (42 %) in the combined 35 mg/m^2^ twice daily cohort had dose reductions by their last treatment cycle, including six patients with a single reduction to 30 mg/m^2^ twice daily and four patients with two dose reductions to 25 mg/m^2^ twice daily. Fourteen patients (58 %) had at least one cycle delayed by ≥4 days (median, two cycles delayed per patient; range 1–4 cycles), and seven patients had delays of ≥8 days (median, one cycle delayed per patient; range 1–2 cycles). Grade 3/4 neutropenia was the most common reason for dose reductions and delays in cycle initiation.

### Safety

Adverse events were observed in all 27 patients. Grade 3/4 toxicity was predominantly hematologic, consisting mostly of neutropenia (70 %) and anemia (22 %) (Table 2). Febrile neutropenia was observed in only two patients (7 %). Prophylactic G-CSF use was not permitted during cycle 1, but was allowed to treat hematologic toxicity according to the American Society of Clinical Oncology guidelines [26]; its use ranged from 20 % of patients (5/25) in cycle 2–67 % of patients (8/12) in cycle 4. Non-hematologic toxicity consisted mainly of fatigue and gastrointestinal disturbances, but rarely was grade 3 or higher (Table 2). Major grade 3/4 toxicities were neutropenia (70 %), anemia (22 %), and leukopenia (19 %). Most of the frequently reported adverse events, including hematologic abnormalities and gastrointestinal disturbances, were considered by the investigator to be related to study treatment.

**Table 2:** Adverse events occurring at incidence ≥20 % or grade 3/4 adverse events occurring at incidence ≥5 % regardless of relation to study treatment

Adverse event, *n* (%)	35 mg/m^2^ (*n* = 24)		All patients (*N* = 27)	
	Any grade	Grade 3/4	Any grade	Grade 3/4
Hematologic				
Neutropenia	19 (79.2)	17 (70.8)	21 (77.8)	19 (70.4)
Anemia	11 (45.8)	6 (25.0)	11 (40.7)	6 (22.2)
Thrombocytopenia	6 (25.0)	1 (4.2)	6 (22.2)	1 (3.7)
Leukopenia	5 (20.8)	4 (16.7)	6 (22.2)	5 (18.5)
Lymphopenia	3 (12.5)	2 (8.3)	3 (11.1)	2 (7.4)
Febrile neutropenia	2 (8.3)	2 (8.3)	2 (7.4)	2 (7.4)
Non-hematologic				
Fatigue	15 (62.5)	0	16 (59.3)	0
Nausea	11 (45.8)	1 (4.2)	13 (48.1)	1 (3.7)
Vomiting	11 (45.8)	1 (4.2)	12 (44.4)	1 (3.7)
Diarrhea	10 (41.7)	1 (4.2)	10 (37.0)	1 (3.7)
Decreased appetite	10 (41.7)	0	10 (37.0)	0
Constipation	6 (25.0)	0	6 (22.2)	0
Blood ALP increased	4 (16.7)	2 (8.3)	4 (14.8)	2 (7.4)
Hyponatremia	4 (16.7)	2 (8.3)	4 (14.8)	2 (7.4)

*ALP* alkaline phosphatase

There were no treatment-related deaths. One patient died of septic shock due to methicillin-resistant *Staphylococcus aureus* during cycle 12, which was considered unrelated to TAS-102 treatment. Serious adverse events occurred in six patients; two had treatment-related hematologic toxicity, and four had adverse events secondary to metastatic disease or other underlying conditions. Two patients discontinued TAS-102 due to adverse events (grade 3 bile duct stenosis and grade 2 diarrhea, respectively), and only one patient discontinued due to a treatment-related adverse event (grade 2 diarrhea).

### Efficacy

**Fig. 2:**
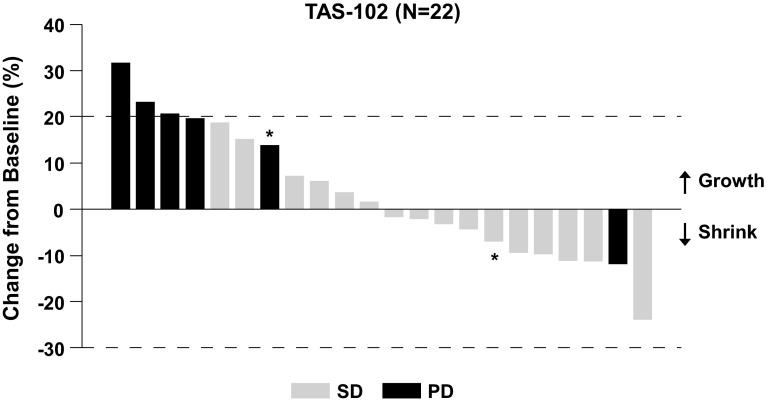
Waterfall plot of target lesion change and best overall response. The *asterisks* denote patients who received TAS-102 at a dose of 30 mg/m^2^ twice daily. Four patients in the efficacy population did not have all target lesions evaluated post-baseline and are excluded from this graph. The second *bar* from the *right* denotes a patient who had progressive disease due to development of a new lesion despite shrinkage of the target lesion

The efficacy evaluation was conducted in 26 patients with post-baseline tumor assessments. Although no patients had a CR or PR, 17 patients achieved an SD lasting for >6 weeks, resulting in a DCR of 65.4 %. Seven of 11 patients with wild-type *KRAS* (64 %) and eight of 13 patients with mutant *KRAS* (62 %) achieved SD. Among the patients receiving 35 mg/m^2^ twice daily (RD level), 16 out of 23 patients achieved an SD, resulting in a DCR of 69.6 % (Fig. 2).

**Fig. 3:**
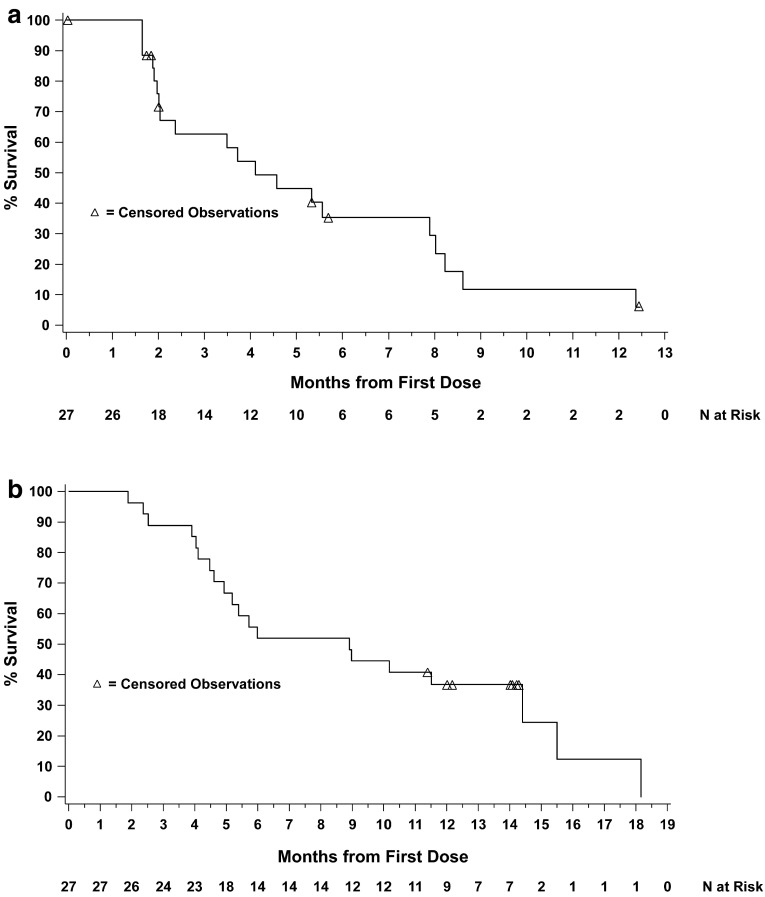
**a** Kaplan–Meier plot of progression-free survival. **b** Kaplan–Meier plot of overall survival

In the entire study population, median PFS and OS were 4.1 months (95 % CI, 2.0–7.9) and 8.9 months (95 % CI, 4.9–14.4), respectively (Fig. 3). Seven patients receiving TAS-102 at a dose of 35 mg/m^2^ twice daily did not have survival events by the data cutoff date and were censored at the date they were last known to be alive. Among the 24 patients receiving 35 mg/m^2^, median PFS and OS were 5.3 months (95 % CI, 2.4–8.0) and 7.5 months (95 % CI, 4.6–14.4), respectively.

Among patients with *KRAS* wild-type and mutant tumors, median PFS was 3.5 months (95 % CI, 2.0–7.9) and 4.1 months (95 % CI, 2.0–8.0), respectively, and median OS was 7.3 months (95 % CI, 4.9–11.5) and 6.0 months (95 % CI, 4.0–14.4), respectively.

## Discussion

In the initial phase 1 trial of TAS-102 in Japanese patients, the RD of 35 mg/m^2^ twice daily on days 1–5 and 8–12 of a 28-day cycle produced a well-tolerated safety profile [22]. Common grade 3/4 adverse events were hematologic, including neutropenia (38 %) and leukopenia (33 %). This was similar to the safety profile observed in previous phase 1 trials in Western patients, though one of these trials conducted in patients with heavily pretreated breast cancer determined an MTD of 25 mg/m^2^ twice daily [19, 20, 23].

Based on preliminary antitumor activity observed in the cohort of patients with colorectal cancer treated in the Japanese phase 1 study (DCR of 50 % [22]), a randomized, double-blind, placebo-controlled, phase 2 trial in Japan was conducted. Patients with metastatic colorectal adenocarcinoma who had received ≥2 prior standard chemotherapy regimens were randomized 2:1 to receive either TAS-102 35 mg/m^2^ or placebo twice daily (days 1–5 and 8–12 of 28-day cycles). Compared with placebo, TAS-102 significantly improved OS (9.0 vs. 6.6 months, HR 0.56, *P* = 0.0011) and PFS with a manageable safety profile [27]. The present trial was designed to confirm the safety of the 35 mg/m^2^ twice daily dose used in the Japanese phase 2 trial in the Western population of patients with colorectal cancer prior to embarking on a global phase 3 trial of TAS-102 in patients with refractory mCRC. In this study, we treated a total of 27 patients, 74 % of whom had received ≥4 prior treatment regimens, in two cohorts: 30 mg/m^2^/dose twice daily and 35 mg/m^2^/dose twice daily. There were no DLTs observed in the first cohort. One patient in the second cohort had dose-limiting grade 3 febrile neutropenia.

Similar to previous studies of TAS-102 conducted in both Japanese and Western populations, grade 3/4 toxicity observed in this trial was predominantly hematologic. Compared with the Japanese randomized phase 2 trial in patients with colorectal cancer, rates of grade 3/4 neutropenia (71 vs. 50 %), febrile neutropenia (8 vs. 4 %), and anemia (25 vs. 17 %) were modestly increased among Western patients, though these findings may be due to the sample size differences between the two trials. In general, toxicities were manageable by appropriate dose delay and/or reduction. Only one patient discontinued study treatment due to a drug-related adverse event. Scheduling prophylactic G-CSF (except during cycle 1) may have avoided dose reductions in some patients who experienced a dose delay. The preliminary efficacy endpoints observed in this phase 1 trial were a DCR of 65 % and a median OS of 8.9 months, which were similar to the results of the Japanese phase 2 trial.

Subsequently, the global phase 3 RECOURSE trial of TAS-102 in patients with refractory mCRC who had at least two prior lines of treatment was initiated based on the RD established in this phase 1 trial. Results from this multicenter trial showed improvement in the primary endpoint of OS compared with placebo [28]. In conclusion, the dose of 35 mg/m^2^ twice daily is generally well tolerated in the treatment of patients with refractory mCRC in a Western population.
